# Time courses of improvement and symptom remission in children treated with atomoxetine for attention-deficit/hyperactivity disorder: analysis of Canadian open-label studies

**DOI:** 10.1186/1753-2000-5-14

**Published:** 2011-05-11

**Authors:** Ruth A Dickson, Ellen Maki, Christopher Gibbins, Stephen W Gutkin, Atilla Turgay, Margaret D Weiss

**Affiliations:** 1 Eli Lilly Canada, Toronto, Canada and University of Calgary, Alberta, Canada; 2 Analytica Statistical Consulting, Don Mills, Ontario, Canada; 3 Children's and Women's Health Centre of British Columbia, Mental Health Research Unit, Vancouver, Canada; 4 Rete Biomedical Communications Corp., Wyckoff, NJ, USA; 5 University of Toronto and Toronto ADHD Clinic, Ontario, Canada

**Keywords:** Attention-deficit/hyperactivity disorder, atomoxetine, drug therapy, remission, response, treatment outcomes

## Abstract

**Background:**

The relatively short durations of the initial pivotal randomized placebo-controlled trials involving atomoxetine HCl for the treatment of attention-deficit/hyperactivity disorder (ADHD) provided limited insight into the time courses of ADHD core symptom responses to this nonstimulant, selective norepinephrine reuptake inhibitor. The aim of this analysis was to evaluate time courses of treatment responses or remission, as assessed by attainment of prespecified scores on the ADHD Rating Scale-IV-Parent Version: Investigator Administered and Scored (ADHDRS-IV-PI) and the Clinical Global Impressions-ADHD-Severity (CGI-ADHD-S) scales, during up to 1 year of atomoxetine treatment in children with ADHD.

**Methods:**

Using pooled data from three Canadian open-label studies involving 338 children ages 6-11 years with ADHD who were treated with atomoxetine for 3, 6 and 12 months, and survival analysis methods for interval-censored data, we estimated the time to: 1) improvement and robust improvement defined by ≥25% and ≥40% reductions from baseline ADHDRS-IV-PI total scores, respectively; and 2) remission using two definitions: a final score of ADHDRS-IV-PI ≤18 or a final score of CGI-ADHD-S ≤2.

**Results:**

The median time to improvement was 3.7 weeks (~1 month), but remission of symptoms did not occur until a median of 14.3 weeks (~3.5 months) using the most stringent CGI-ADHD-S threshold. Probabilities of robust improvement were 47% at or before 4 weeks of treatment; 76% at 12 weeks; 85% at 26 weeks; and 96% at 52 weeks. Probabilities of remission at these corresponding time points were 30%, 59%, 77%, and 85% (using the ADHDRS-IV scale) and 8%, 47%, 67%, and 75% (using the CGI-ADHD-S scale). The change from atomoxetine treatment month 5 to month 12 of -1.01 (1.03) was not statistically significant (*p *= .33).

**Conclusions:**

Reductions in core ADHD symptoms during atomoxetine treatment are gradual. Although approximately one-half of study participants showed improvement at 1 month of atomoxetine treatment, remission criteria were not met until about 3 months. Understanding the time course of children's responses to atomoxetine treatment may inform clinical decision making and also influence the durations of trials comparing the effects of this medication with other ADHD treatments.

**Trial Registrations:**

clinicaltrials.gov: NCT00191633, NCT00216918, NCT00191880.

## Background

Both psychostimulants and the selective norepinephrine reuptake inhibitor atomoxetine HCl are recommended psychopharmacological treatment options for children diagnosed with attention-deficit/hyperactivity disorder (ADHD), which is considered to be the most common neurobehavioral disorder affecting children [1-3]. In clinical practice, the onset of efficacy and times to symptom improvement and remission during atomoxetine treatment are different from those of stimulant medications, leading to questions about the time required to optimize atomoxetine treatment responses.

Our initial understanding of the time courses of atomoxetine responses was based on randomized placebo-controlled clinical trials with relatively short durations (typically ≤9 weeks) [4-8]. Notably, in these pivotal trials symptom scores appeared to be still descending (improving) at study completion. Hence, it was not possible to determine conclusively from these trials if ADHD core symptoms could continue to decrease, and if so, how quickly (or slowly) and to what extent. These issues have implications for the evolving discussion about ADHD symptom remission as a treatment goal; that is, the concept that the target of ADHD treatment should be minimal or no symptoms, a loss of diagnostic status, and optimal functioning [9,10].

Although attainment of predefined thresholds on validated scales as a measure of symptom remission is a useful barometer of improvement, the time courses of responses to various treatments must be considered when this outcome measure is used to compare interventions; this may be of marked importance when comparing treatments for ADHD--both pharmacological and non-pharmacological (e.g. behavioral and psychoeducational interventions)--that have slower onsets of actions compared with stimulants. Stimulants are notable within the psychopharmacological armamentarium for the relative short time to peak clinical effects.

Improvements in ADHD symptoms have been defined as ≥25% reductions (and robust improvement as ≥40% reductions) on the ADHD Rating Scale-IV-Parent Version: Investigator Administered and Scored (ADHDRS-IV-PI) total score [4-11]. However, response definitions based on percentage reduction in scale scores do not take into account baseline symptom severity; for children with very severe disease, robust changes may represent substantial improvements yet leave them very impaired, whereas children with less severe disease who just meet diagnostic criteria may attain normalization for age and gender after only modest percentage reductions in core symptoms. It is therefore helpful for interpreting symptomatic outcomes also to define symptomatic remission. Operational definitions of symptomatic remission include: 1) an ADHDRS-IV-PI total score of ≤18 (average per-item score of ≤1), where 0 signifies "not [no symptoms] at all" and 3, "very much"; and 2) a Clinical Global Impressions-ADHD-Severity (CGI-ADHD-S) scale score of ≤2, where 1 signifies "not at all ill," 2 "minimally ill," and 7 "maximal, profound impairment" [4-12].

The primary objective of this study was to determine times to response and remission according to predefined thresholds on the ADHDRS-IV-PI and CGI-ADHD-S scales in children treated with atomoxetine at usual clinical dosages [11,12]. To accomplish this aim, we estimated the likelihood of response or remission with atomoxetine as a function of time using pooled data from three Canadian clinical trials with durations of up to 1 year [13-15]. Equipped with a more detailed and nuanced understanding of the time course of treatment responses and remission with atomoxetine, clinicians may be better able to: 1) educate (and calibrate the expectations of) children and their parents/guardians/teachers concerning time courses to different levels of response or remission; 2) decide how long to continue a medication trial; and/or 3) determine if (and when) treatment augmentation or alteration might be needed. By understanding the time courses of response and remission, ADHD researchers may also be better positioned to design more clinically meaningful trials comparing the effects of atomoxetine, stimulants, and/or other treatments.

## Methods

### Overview of studies analyzed

In this retrospective efficacy analysis, data were pooled from three Canadian open-label studies [13-15]. These trials assessed the effects of atomoxetine on both core symptoms and a broad range of other outcome measures (the latter are not considered in this report). The duration of treatment was 3 months in Study S012, 6 months in Study S013, and 1 year in Study LYCS.

### Study Participants

Outpatient boys and girls aged 6 to 11 years (n = 212 in Study S012; n = 21 in Study S013; n = 105 in Study LYCS) who had a diagnosis of ADHD according to the *Diagnostic and Statistical Manual of Mental Disorders Text Revision *(*DSM-IV-TR) *[16] were eligible. Minimum symptom severity, as measured by the ADHDRS-IV-PI total score, was ≥1.5 standard deviations (*SD*) above age and gender norms. A slight difference between trials was in study participant age ranges at entry: 6 to 11 years in Study S012, 6 to 10 years in Study S013, and 8 to 11 years in Study LYCS. All children had normal intelligence based on investigator judgment and a score of ≥85 if formal IQ testing was conducted.

Exclusion criteria included a history of bipolar disorder, psychosis, pervasive developmental disorders, conduct disorder, seizure disorder (other than febrile seizures), or serious suicide risk. Eligible study participants also were not using other psychotropic medications and had no medical condition that would contraindicate the use of atomoxetine.

### Study designs

Study participant data were collected at child outpatient clinics in Canada from August 2004 through June 2006. Investigators were child psychiatrists or pediatricians. All studies conformed to ethical principles of the Declaration of Helsinki and all applicable laws, regulations, and good clinical practices, and were approved by ethics committees. Written informed consent was obtained from parents/legal guardians and assent from children. All eligible study participants completed baseline assessments.

In Study LYCS, which commenced before atomoxetine had been approved by Health Canada, atomoxetine treatment was started at 0.5 mg/kg/day for the first 7 days, then increased to approximately 1.2 mg/kg/day. An additional increase to the maximum of 1.4 mg/kg/day or 100 mg/day (whichever was less) based on efficacy and tolerability profiles was allowed [13]. In Studies S012 and S013 [14,15], atomoxetine was titrated more slowly (according to the approved Canadian atomoxetine product monograph), to a maximum of 1.4 mg/kg/day as follows: 0.5 mg/kg/day for the first 10 days; 0.8 mg/kg/day over the next 10 days; and 1.2 mg/kg/day for a minimum of 10 days, with an increase to 1.4 mg/kg/day allowed thereafter. All study participants were atomoxetine-naïve at study entry. Studies also differed in study participants' prior use of stimulants for ADHD. In Study LYCS, all children entering were required to be stimulant-naïve, whereas both stimulant-naïve and stimulant-treated children were eligible for Study SO12 and Study SO13. Use of psychotropic medications other than atomoxetine was not permitted in any of the studies.

### Measures

Efficacy measures in the present study were the ADHDRS-IV-PI and the CGI-ADHD-S [11,12]. The timing of assessments varied across the studies. The ADHDRS-IV-PI was assessed at baseline in all three studies and at: 1) months 1, 3, 5, 8, and 12 in Study LYCS; 2) months 1, 2, and 3 in Study S012; and 3) months 2 and 6 in Study S013. In all three studies, the CGI-ADHD-S was assessed at baseline and again at week 2 with further assessments at: 1) months 1, 2, 3, 4, 5, 6, 8, 10, 12 in Study LYCS; 2) week 3 and months 1, 2, and 3 in Study S012; and 3) week 3 and months 1, 2, 4, and 6 in Study S013.

Symptom improvement was operationally defined *a priori *as a ≥25% decrease from baseline on the ADHDRS-IV-PI, and robust improvement as a ≥40% decrease. Remission was defined in two ways: by a threshold ADHDRS-IV-PI score ≤18 or a CGI-ADHD-S score ≤2.

### Statistical Analyses

Responses noted for the first time at a given visit are unlikely to have occurred precisely at the moment of the assessment; we can most accurately state that the response occurred at some time between the current and the most recent previous visit. In such cases, the data are said to be "interval-censored" [17]. Some study participants had not achieved a response by the time of their final assessment; therefore, the time between baseline and their last assessment served as a lower bound on the time required to attain a response. Such data are said to be "right-censored" [18]. Our pooled dataset contained both interval- and right-censored data.

Survival analysis techniques, which utilize data from all study participants to the point that they either experience a response or are no longer available to be followed, are the most appropriate methods for handling censored data where the endpoint is the time to an event of interest. Such methods allow data contributed by study participants who withdrew early, or completed the study but did not respond, to be included in the analysis. These methods could accommodate data from all three studies, regardless of the fact that the assessment times varied between studies, and could also allow inclusion of data from unscheduled visits and visits that occurred outside of prescribed visit windows.

Because the time necessary to realize each of the four treatment response criteria was of interest in the current investigation, survival analysis methods were used. Turnbull's extension of the Kaplan-Meier curve to interval-censored data was used to estimate the cumulative probability of response over time (survivor function) as well as to estimate the median time to response [17]. A log-normal parametric model was used to assess the univariate impact of baseline ADHDRS-IV-PI and baseline CGI-ADHD-S on the time to each of the four endpoints of interest. A repeated-measures linear mixed model was used to obtain the least-squares mean of ADHDRS-IV-PI by month.

## Results

### Baseline characteristics

The pooled study sample comprised 249 (73.7%) boys and 89 (26.3%) girls whose mean (standard error [*SE*]) age (overall) was 8.7 (0.08) years. Of study participants in Study S012, 54.7% were stimulant-naïve compared with 38.1% of those in Study S013 and 100% of those in Study LYCS. Mean (*SE*) baseline scores were 39.1 (0.43) on the ADHDRS-IV-PI and 4.8 (0.05) on the CGI-ADHD-S (Table 1).

**Table 1:** Summary of Baseline Characteristics

Characteristic	Study			
	
	LYCS (n = 105)	S012 (n = 212)	S013 (n = 21)	All (N = 338)
Gender, male, n (%)	76 (72.4)	157 (74.1)	16 (76.2)	249 (73.7)
Age	9.3 (0.08)	8.5 (0.11)	8.0 (0.28)	8.7 (0.08)
ADHD subtype, n (%)				
Combined	74 (70.5)	166 (78.3)	6 (28.6)	246 (72.8)
Inattentive	31 (29.5)	43 (20.3)	15 (71.4)	89 (26.3)
Hyperactive/impulsive	0 (0.0)	3 (1.4)	0 (0.0)	3 (0.9)
Stimulant-naïve, n (%)	105 (100.0)	116 (54.7)	8 (38.1)	229 (67.8)
ADHDRS-IV-PI	37.0 (0.84)	40.5 (0.52)	35.9 (1.13)	39.1 (0.43)
CGI-ADHD-S	4.6 (0.08)	4.8 (0.06)	4.9 (0.10)	4.8 (0.05)

ADHDRS-IV-PI = Attention-Deficit/Hyperactivity Disorder Rating Scale Parent Version: Investigator Administered and Scored; CGI-ADHD-S = Clinical Global Impression of ADHD Severity.

Mean (*SE*) or numbers (%).

### Subject disposition

Approximately 70% of study participants completed each study: 68.6% in LYCS, 70.8% in S012, and 76.2% in S013.

The most common reason for discontinuation in all studies was lack of efficacy as perceived by the child/caregiver or physician (10.7%), followed by adverse events (6.5%). Five (1.5%) study participants were lost to follow-up.

### Outcomes

The median number of weeks from baseline to the last available ADHDR-IV-PI assessment for the pooled studies was 13 weeks (~3 months). The follow-up time between the baseline and last available assessment varied according to the duration of each study. Table 2 reports the mean (*SE*) and median (range) duration of follow-up (weeks) by individual study, as well as for the combined studies.

**Table 2:** Summary of Subject Follow-up

	Study			
	
	LYCS	S012	S013	All
**Weeks From Baseline to Last ADHDRS-IV-PI Assessment**				
N†	102	198	20	320
Mean (*SE*)	42.7 (1.63)	11.5 (0.23)	22.5 (1.64)	22.2 (0.97)
Median	51.3	12.9	25.9	13.3
Range	3.9-58.0	1.0-17.3	4.4-27.1	1.0-58.0
**Weeks From Baseline to Last CGI-ADHD-S Assessment**				
N†	102	206	21	329
Mean (*SE*)	43.1 (0.15)	11.2 (0.25)	21.5 (1.9)	21.7 (0.96)
Median	51.3	12.7	25.9	13.3
Range	2.3-58.0	1.0-17.3	1.6-27.1	1.0-58.0

†Only study participants with both a baseline value and at least one post-baseline value were included in this summary.

ADHDRS-IV-PI = Attention-Deficit/Hyperactivity Disorder Rating Scale Parent Version: Investigator Administered and Scored; CGI-ADHD-S = Clinical Global Impression of ADHD Severity.

Atomoxetine treatment reduced ADHD symptoms sharply during the first month and was associated with further reductions in subsequent months. Mean ADHDRS-IV-PI total scores leveled out at approximately month 5 (Table 3). The change from atomoxetine treatment month 5 to month 12 of -1.01 (1.03) was not statistically significant (*p *= .33).

**Table 3:** ADHDRS-IV-PI Least-Square (LS) Mean by Month of Atomoxetine Treatment

Time Point	N	LS Mean (*SE*)
Day 0	335	39.12 (0.43)
Month 1	282	22.88 (0.65)
Month 2	179	21.00 (0.67)
Month 3	176	19.93 (0.67)
Month 5	85	17.83 (0.85)
Month 6	21	17.48 (1.79)
Month 8	81	17.27 (0.92)
Month 12	72	16.82 (0.99)

ADHDRS-IV-PI = Attention-Deficit/Hyperactivity Disorder Rating Scale Parent Version: Investigator Administered and Scored.

As shown in Table 4, the median time to treatment response varied markedly by criterion. The median time to improvement according to the least stringent criterion of treatment response was 3.7 weeks; the probability of improvement was estimated at 60% at or before 4 weeks and 88% at or before 12 weeks. The median time to a robust improvement was slightly greater (4.7 weeks), whereas the estimated probability of a robust improvement was 47% at or before week 4 and 76% at or before week 12 (Table 4).

**Table 4:** Response Probabilities Over Time

Endpoint	Probability That Endpoint Was Observed at or Before:				Expected Week by Which This Percentage of Study Participants Will Have Achieved Endpoint:		
	
	4 weeks	12 weeks	26 weeks	52 weeks	25%	50%	75%
Improvement	0.60 (0.38, 0.79)	0.88 (0.75, 0.95)	0.96 (0.85, 0.99)	1.00	--	3.7	7.2
Robust Improvement	0.47 (0.30, 0.65)	0.76 (0.60, 0.87)	0.85 (0.67, 0.94)	0.96 (0.87, 0.99)	3.6	4.7	12.0
Remission*	0.30 (0.16, 0.49)	0.59 (0.34, 0.79)	0.77 (0.48, 0.92)	0.85 (0.72, 0.93)	3.7	8.0	23.8
Remission**	0.08 (0.04, 0.16)	0.47 (0.26, 0.70)	0.67 (0.51, 0.80)	0.75 (0.60, 0.85)	5.3	14.3	52.3

**^†^**Probability with 95% confidence interval.

Remission using two definitions: *final score of ADHDRS-IV-PI ≤18, and **CGI-ADHD-S ≤2. ADHDRS-IV-PI = Attention-Deficit/Hyperactivity Disorder Rating Scale Parent Version: Investigator Administered and Scored; CGI-ADHD-S = Clinical Global Impression of ADHD Severity.

Longer treatment intervals were required to meet definitions of symptom remission. The median time to remission, defined as a total ADHDRS-IV-PI of ≤18, was 8.0 weeks, and the probability of remission did not reach 75% until approximately 24 weeks. To meet the most rigorous criterion for remission (achieving a CGI-ADHD-S score ≤2), the median time was 14.3 weeks. For this endpoint, the probability of remission by 26 weeks was approximately 67% (Table 4).

**Figure 1:**
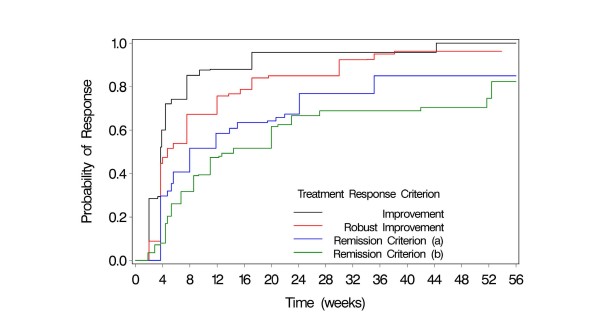
**Response Probabilities Over Time**. Response using two definitions: improvement = ≥25% reduction from baseline on the ADHDRS-IV-PI and robust improvement = ≥40% reduction. Remission using two definitions: a final score of (a) ADHDRS-IV-PI ≤18 or (b) CGI-ADHD-S ≤2. ADHDRS-IV-PI = ADHD Rating Scale Parent Version: Investigator Administered and Scored; CGI-ADHD-S = Clinical Global Impressions-ADHD-Severity.

Figure 1 presents the estimated cumulative probability (Turnbull estimate) of meeting the response/remission criteria at or before each month of atomoxetine treatment; the curves for each of the four criteria are shown. For each of the four treatment response criteria, the estimated probability of achieving the endpoint at or before week 16 of atomoxetine treatment was at least 50%, and by week 20 it was at least 60%.

On univariate analysis, the baseline value of ADHDRS-IV-PI total score was a significant predictor of the time to improvement, robust improvement, and an ADHDRS-IV-PI ≤18, but not of a CGI-ADHD-S score of ≤2. Higher baseline ADHDRS-IV-PI scores (indicative of more severe ADHD symptomatology) were associated with a shorter time to improvement and robust improvement; whereas lower baseline ADHDRS-IV-PI total scores were associated with a shorter time to attain remission. The baseline value of CGI-ADHD-S was also a significant predictor of time to remission, with lower values (indicative of less severe ADHD symptomatology) being associated with shorter time required to achieve remission defined as a CGI-ADHD-S score of ≤2.

## Discussion

In this study, there was a progressive increase in the likelihood that treatment met predefined thresholds for response and remission with increasing time on atomoxetine. Of interest, children's ADHD symptoms continued to improve up to approximately 5 months. Although there was symptomatic improvement as measured by the ADHDRS-IV-PI total score beyond this time, the change was not statistically significant.

The present analysis of three Canadian open-label clinical trials of atomoxetine produced clinically meaningful findings that are consistent with data from two recently reported European randomized, double-blind, placebo-controlled studies that reported increased efficacy of atomoxetine over time [19,20]. First, a Spanish study of 151 treatment-naïve children and adolescents (mean age = 10.3 years) with newly diagnosed ADHD showed that reductions (improvements) in the ADHDRS-IV-PI total score in the atomoxetine (vs. placebo) group first reached statistical significance at treatment week 4; of importance, statistically significant reductions in ADHD symptoms were evident between weeks 6 and 12 [19]. Similarly, in a 10-week Swedish study involving 99 stimulant-naïve children and adolescents (mean age = 11.5 years), mean changes in the ADHDRS-IV-PI total score from baseline in study participants receiving atomoxetine in combination with psychoeducation (vs. placebo in combination with psychoeducation) reached statistical significance at treatment week 3 and continued to improve at each subsequent visit [20].

The gradual and progressive efficacy of atomoxetine in treating core symptoms of ADHD, which was observed in the present study, has potential implications for understanding three previously reported studies comparing the effects of atomoxetine to those of long-acting stimulants [21-23]. The Strattera Adderall XR Randomized Trial (St.A.R.T) was an analog classroom study designed to assess the time course of treatment effect, tolerability, and safety of mixed amphetamine salts extended-release (MAS XR) compared with atomoxetine in children with ADHD. The randomized double-blind treatment period was 18 days. The authors concluded that the difference in effect size in this study indicated a more robust treatment effect of MAS XR [23]. The open-label study by Kemner and co-workers (2005) concluded that treatment with osmotic-release oral system methylphenidate (OROS MPH) exerted greater effects on core ADHD symptoms compared with atomoxetine; however, this was a short-term study (3 weeks) [22]. A 6-week active-comparator study also found that OROS MPH was superior to atomoxetine for the total group, but there were no differences in response rates for the treatment-naïve subgroup [21].

Evidence from these comparator studies, the European studies discussed above [19,20], and our analysis supports the clinical perception that atomoxetine is not the ideal treatment for patients who require rapid control of symptoms. However, it is potentially misleading to compare effects of long-acting stimulants to those of atomoxetine based on short-term studies because atomoxetine requires a longer treatment period to: 1) attain an initial response and 2) achieve maximal possible reductions in ADHD symptoms. Long-term direct comparison studies of atomoxetine and stimulants are necessary to determine if the patterns of symptom improvement with atomoxetine treatment differ from those with stimulants in the long term as well as the short term.

In our study, baseline illness severity substantially influenced the time to meet predefined measures of improvement. Children initiating atomoxetine therapy with higher symptom severity scores met percentage improvement criteria in a shorter time than children starting with lower baseline symptom severity scores. In contrast, children rated as less severely symptomatic on the CGI-ADHD-S and ADHDRS-IV-PI achieved remission thresholds more rapidly. Of clinical interest, the Integrated Data Exploratory Analysis study, a retrospective analysis of randomized controlled trials having 6- to 9-week durations, did not identify potential baseline (moderator) predictors of response but did find that the only predictor (on-treatment mediator) of a much improved response at trial endpoint (≥40% decrease on the ADHDRS-IV-PI) was being at least minimally improved (≥25% but <40% decrease on the ADHDRS-IV-PI) by treatment week 4 [24]. This finding of improvement at 4 weeks' predicting later improvement, taken together with the possibility of continued improvement over time (as found in the present Canadian and European studies [13-15,19,20]), suggests that monitoring for improvement at 1 month is valuable, as is continued monthly monitoring to confirm the presence of progressive improvement in ADHD symptoms that would justify continuation of atomoxetine treatment.

## Limitations

Our findings are most generalizable to Canadian children ages 6 to 11.5 years who: 1) are of at least average intelligence and whose ADHD symptoms are well above age and gender norms for severity; 2) do not have comorbid conditions such as bipolar disorder, psychoses, pervasive developmental disorders, conduct disorder, or serious suicide risk; and/or 3) are treated with atomoxetine on an outpatient basis. Most study participants (>70%) were boys and had the combined subtype of ADHD. In addition, approximately two-thirds of enrolled children (and all participants in the 1-year study) were previously untreated with ADHD medications and therefore may have been more responsive to medication than children who had received, but failed, prior treatments. The trials were all open label, and this study design feature may have encouraged positive clinician bias in estimating improvements; in Canada, it was considered unethical to include a placebo arm in extended-duration studies of ADHD. The results of the three open-label trials included in our study may have been influenced by initial patient selection and rater drift. Given the characteristics of our sample, the designs of the studies, and the potential advantages to children of clinical trial participation, it is possible that the relatively high rates of response we report with atomoxetine treatment will not be replicated in usual clinical practice.

The slow upward titration of atomoxetine and the differences in the titration schedules mandated by the study protocols may have had some impact on early response rates, but it is unlikely that the dosing of atomoxetine during the initial part of the study impacted the major finding of the study, which was that there is slow but progressive improvement over time during atomoxetine treatment long after titration is complete.

Only one of the primary studies (Study SO13) analyzed herein included teachers' evaluations, precluding inclusion of teachers' important perspectives on time courses to response or remission with atomoxetine. In Study S013, baseline symptom ratings completed by the teachers were indicative of less severe symptoms than ratings completed by parents, but the progressive improvements during this 6-month study showed that symptom decrements noted by parents paralleled those of teachers [14]. In addition to including data from a limited range of informants, our analyses were limited to measures of core ADHD symptoms. Achieving a pre-defined 'remission' score on the CGI-ADHD-S is indicative of improvement in ADHD symptomatology but does not necessarily mean that the child is without impairments. Future clinical trials, especially those comparing different treatments, should evaluate times to achievement of thresholds or norms on other important measures, including health-related quality-of-life and functional/cognitive/neuropsychological outcomes.

Not all subjects had 12 months of follow-up; the three studies varied in length from 3-12 months and in addition approximately 30% of children did not complete their study. Therefore, we chose methods of analysis (survival curves) that do not require an equal length of follow-up for each subject and that did not require that all subjects complete the study. These methods estimate response probabilities over time by using the data for each subject up to the point that they either responded or withdrew from the study. We don't know the time by which the drop-outs would have responded but each participant contributes information: for example, a child who discontinued at week 6 prior to responding provides information on the probability of achieving (or not achieving) a response by week 6.

When interpreting such curves, it is essential to understand both that there are declining numbers of participants over time, and that the cumulative response probability at a given time point is based not only on those subjects still under observation, but also on the pattern of response and withdrawals at all previous time points. Thus the response probability at a certain time depends directly on those still under observation, and indirectly on those who withdrew or responded earlier.

## Conclusions

Our findings concerning time courses of response and remission with atomoxetine may inform future study design and clinical decision making. From a clinical perspective, appropriate expectations should be set with children and parents at the time of atomoxetine initiation, because reductions in symptoms are gradual but appear to progress over time. Designs of future comparison studies of medications to treat ADHD should consider differences in time of onset of efficacy (and time to peak response) between atomoxetine and stimulants and/or other treatments.

## Abbreviations

ADHD: attention-deficit/hyperactivity disorder; ADHDRS-IV-PI: ADHD Rating Scale Parent Version: Investigator Administered and Scored; CGI-ADHD-S: Clinical Global Impressions-ADHD-Severity; MAS XR: mixed amphetamine salts extended-release; OROS MPH: osmotic-release oral system methylphenidate;

## Competing interests

RAD: employee of the study sponsor with stock or equity >$10,000. EM: paid consultant to the study sponsor. CG: no competing interests to disclose. SWG: paid consultant to the study sponsor and its affiliates, as well as BioBehavioral Diagnostics. MDW: advisory boards and speakers' bureaus, consultant, grant and/or other research support, study sponsor, Abbott, Janssen, Purdue Pharma, Shire, Takeda; speakers' bureau, Novartis; travel support from study sponsor to present poster at congress.

## Authors' contributions

All authors contributed to the conception and design of the study.

MDW, AT, and other clinical investigators acquired data. EM conducted the statistical analysis. All authors interpreted the data. RAD, EM and SWG wrote the manuscript, and all authors contributed to the manuscript. All have read and approved the final manuscript except for AT. Dr. Atilla Turgay (deceased) participated in the data analysis and an early draft of the manuscript. RAD had access to all data and takes responsibility for the study and its report (study guarantor).
